# Dynamic glycosylation remodeling in neurological disorders

**DOI:** 10.3389/fnmol.2025.1674665

**Published:** 2025-12-02

**Authors:** Dan Xing, Yingxun Gong, Weiyi Xia, Huifang Tu, Limei Yuan, Yiqing Yin, Kaiyuan Wang

**Affiliations:** 1 Tianjin Medical University, Tianjin, China; 2 State Key Laboratory of Druggability Evaluation and Systematic Translational Medicine, Department of Anesthesiology, Tianjin’s Clinical Research Center for Cancer, National Clinical Research Center for Cancer, Tianjin Medical University Cancer Institute and Hospital, Tianjin, China; 3 Graduate School, Tianjin University of Traditional Chinese Medicine, Tianjin, China

**Keywords:** glycosylation, O-GlcNAcylation, neurodegeneration, psychiatry, Alzheimer’s disease

## Abstract

Glycosylation, a crucial post-translational modification, involves the covalent attachment of monosaccharides or oligosaccharides to proteins. This process significantly influences protein stability and function. Within the nervous system, glycosylation regulates key processes including neuronal differentiation, migration, synapse formation, and neurotransmitter release and signaling. Its proper functioning is essential for maintaining neuronal homeostasis and reducing the risk of neurological disorders. Understanding the specific mechanisms by which glycosylation impacts the central nervous system is therefore essential for developing novel therapeutic strategies. This review focuses on the roles of three major glycosylation types–N-glycosylation, O-glycosylation, and O-GlcNAcylation–in the pathogenesis of central nervous system disorders.

## 1 Introduction

The central nervous system (CNS) is comprised of the brain and spinal cord, and serves as the command central for essential functions, including cognition, emotion, motor control, and sensory processing. Dysregulation within the CNS has been demonstrated to result in a range of pathologies, including neurodevelopmental, neurodegenerative, and neuropsychiatric conditions, which have been shown to have a profound impact on quality of life.

Protein glycosylation refers to the enzymatic process whereby saccharides are covalently attached to proteins, influencing their stability, localization, and function (Eichler, 2019). Among the diverse forms of glycosylation, N-glycosylation, O-glycosylation, and O-GlcNAcylation are particularly relevant to disease mechanisms. These modifications have been implicated in various pathologies including cancer, immune dysregulation, metabolic syndromes, and neurological disorders (He et al., 2022; Memarian et al., 2021; Wolters-Eisfeld et al., 2018; Yun et al., 2023). Beyond protein glycosylation, glycoconjugates such as glycosphingolipids (a major class of glycolipids) and glycosaminoglycans (GAGs) also play critical roles in CNS development and function (Ali et al., 2025). Glycosphingolipids, including gangliosides, are integral components of the plasma membrane and are vital for cell recognition, signal transduction, and modulating neuronal integrity (Ali et al., 2025; Schnaar et al., 2014). GAGs, which are long, linear polysaccharides often covalently linked to core proteins to form proteoglycans, are ubiquitous constituents of the extracellular matrix (ECM) and cell surfaces. They are involved in regulating neural cell migration, axon guidance, synaptogenesis, and are implicated in the response to neural injury through their influence on neuroinflammation and ECM remodeling (Ali et al., 2025; Schengrund, 2015).

This review examines how specific glycosylation pathways–N-glycosylation, O-glycosylation, and O-GlcNAcylation–contribute to the pathogenesis of CNS disorders. By elucidating the molecular mechanisms underlying glycosylation-related dysfunction, we aim to highlight potential diagnostic and therapeutic strategies that may ultimately improve patient outcomes.

## 2 Neurodevelopmental diseases

**FIGURE 1:**
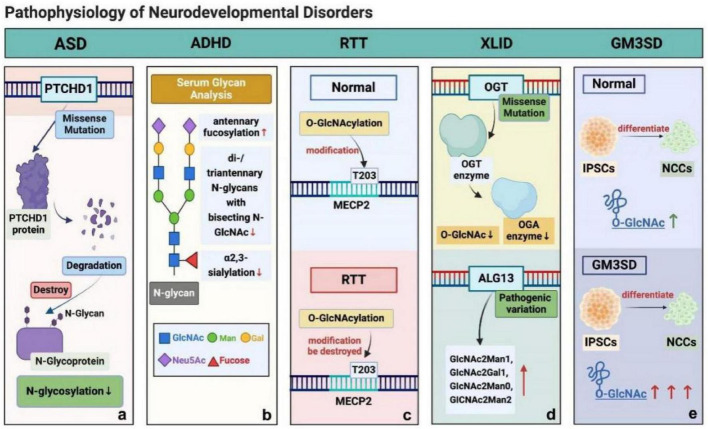
Pathophysiological role of glycosylation in neurodevelopmental disorders. **(a)** PTCHD1 missense mutations produce unstable proteins prone to misfolding and degradation, impairing N-glycosylation. **(b)** ADHD patients exhibit elevated antennal fucosylation in serum, alongside reduced di-/triantennal N-glycans containing GlcNAc and α2,3-sialylation. **(c)** In RTT, disrupted O-GlcNAcylation at MECP2 T203 is a key pathogenic event, whereas this modification occurs dynamically under normal conditions. **(d)** XLID-linked OGT catalytic domain mutations lower OGT activity, decreasing O-GlcNAc and OGA levels; ALG13 variants also impair N-glycosylation in XLID. **(e)** During neural crest cell differentiation, GM3 synthase-deficient iPSCs show broader O-GlcNAc distribution, indicating GM3 loss perturbs neuro-specific glycolipid glycosylation.

Neurodevelopmental disorders constitute a multifaceted cohort, arising during the crucial developmental phase of life. Typically, before formal schooling, they are characterized by early onset and profound deficiencies in personal, social, academic, and vocational functioning, as outlined in the Diagnostic and Statistical Manual of Mental Disorders, Fifth Edition (DSM-5) and the International Classification of Diseases, 11th Revision (ICD-11). Neurodevelopmental disorders span a vast array, prominently featuring autism spectrum disorder (ASD), attention deficit hyperactivity disorder (ADHD), and intellectual disability (ID)–a category which includes etiologically distinct entities such as X-linked intellectual disability (XLID) and Rett syndrome. Furthermore, certain inborn errors of metabolism, such as GM3 synthase deficiency, are also discussed herein due to their profound and characteristic neurodevelopmental phenotypic presentations. These conditions can present intricate challenges across multiple domains of development, including cognition, behavior, and social interaction (Figure 1).

Neurodevelopmental disorders have a multifactorial etiology, arising from a combination of genetic predispositions (Parenti et al., 2020) and a range of prenatal environmental risk factors (Doi et al., 2022). These include maternal immune activation, which has been demonstrated to perturb fetal neuroimmune programming (Estes and McAllister, 2016). Furthermore, exposure to teratogenic substances such as valproic acid, a known antiepileptic agent, has been shown to be significantly associated with an elevated risk of autism spectrum disorders in offspring (Christensen et al., 2013; Kataoka et al., 2013). Additionally, complications such as preterm birth and low birth weight (Singh et al., 2013) have been observed to be associated with impaired neuromaturation. Collectively, these factors represent significant gestational adversities with the potential to alter typical neural developmental trajectories.

### 2.1 Autism spectrum disorder

Autism spectrum disorder (ASD) consists of a group of heterogeneous genetic neurobehavioral conditions, characterized by core symptoms including impairments in social communication, repetitive and stereotyped behaviors, and abnormal sensory responses–such as excessive or diminished reactivity to auditory, tactile, or visual stimuli (Genovese and Butler, 2023). ASD is strongly influenced by genetic factors, with well-documented contributions from both monogenic variants (e.g., FMR1, SHANK3, TSC1/2, MECP2) and chromosomal copy number variants (e.g., deletions at 15q11-q13, deletions/duplications at 16p11.2, and deletions at 22q11.2) (Francisco et al., 2020; Loureiro et al., 2021; Lyons-Warren et al., 2022; Philippe, 2022; Varghese et al., 2017; Villa et al., 2021; Zhang W. J. et al., 2022). Beyond genetics, environmental factors have also been linked to ASD risk, including prenatal exposure to valproic acid (VPA) (Danzer, 2019), pregnancy infection (Tioleco et al., 2021), maternal obesity (Tong and Kalish, 2021), gestational diabetes (Xiang et al., 2015).

In addition to these well-established genetic and environmental drivers, recent research has identified more genetic contributors to ASD pathogenesis. For instance, genes such as PTCHD1 are now implicated in increasing ASD susceptibility. It is hypothesized that PTCHD1 exerts its function through mechanisms associated with glycosylation. PTCHD1 is a gene associated with intellectual disability and autism spectrum disorder. Specific missense mutations in PTCHD1 (such as those identified in patient-derived variants, including p.L336P, p.C384Y, and others) lead to the production of unstable proteins that fail to achieve proper folding, resulting in their degradation. These mutations exert pathogenic effects primarily by reducing protein stability and impairing N-glycosylation, rather than by disrupting the trafficking of PTCHD1 to its functional locations (Xie et al., 2024).

### 2.2 Attention deficit/hyperactivity disorder

Attention deficit/hyperactivity disorder (ADHD) is a heterogeneous and highly heritable disorder that commonly occurs in school-aged children. In addition to the learning difficulties associated with inattention and the core symptoms of ADHD, children with ADHD also exhibit hyperactivity and impulsivity (Posner et al., 2020). The exact causes and risk factors for ADHD are unknown. However, many studies have identified risk factors associated with ADHD, such as genetics, alcohol use or smoking during pregnancy, and child health conditions, including head injuries, parental mental health, and the family environment (Bitsko et al., 2024; Claussen et al., 2024; Dimitrov et al., 2024; Faraone et al., 2021; Maher et al., 2024; Robinson et al., 2024; So et al., 2024).

Traditionally, the diagnosis of ADHD is mostly based on the clinical presentation, description of the patient’s parents, questionnaires, rating scales, and screening tests (Bélanger et al., 2018). Recently, several studies have investigated molecular and biochemical markers in the serum of ADHD patients with the goal of identifying new biomarkers of ADHD. Kianičková et al. (2023) utilized serum glycan analysis and demonstrated that antennary fucosylation was upregulated, while di-/triantennary N-glycans containing bisecting N-acetylglucosamine (GlcNAc) and α2,3-sialylation were reduced in the serum of patients with ADHD. As major types of glycan processing, core fucosylation (catalyzed by FUT8) and sialylation (mediated by sialyltransferases) represent key terminal modifications that determine the structural and functional diversity of glycoconjugates, and their dysregulation is a hallmark in various disease states (Schjoldager et al., 2020). Although the sample size and design of this study were insufficient to draw broad conclusions, the above results may provide new perspectives for studying the functional associations of altered glycosylation in ADHD and suggest that the pathogenesis of ADHD may be related to glycosylation (Kianičková et al., 2023).

### 2.3 Rett syndrome

Rett syndrome (RTT) is classified within autism spectrum disorders (Percy, 2011), and it is defined by the regression of purposeful hand use and spoken language, with the development of gait abnormalities and hand stereotypies. After the period of regression, a stage of stabilization and potentially even improvement ensues, with some individuals partially regaining skills (Neul et al., 2010). Most cases of RTT are caused by *de novo* heterozygous pathogenic loss-of-function variants in the X-linked transcriptional regulator methyl-CpG binding protein 2 (MECP2). A previous study revealed the dynamic modification of MeCP2 at threonine 203 (T203), a causative site in RTT, by O-linked-β-N-acetylglucosamine (O-GlcNAc). Disruption of O-GlcNAcylation specifically at T203 hindered dendrite growth and spine maturation in hippocampal neurons and disrupted neuronal migration and dendritic spine development, leading to impairments in synaptic transmission in developing and juvenile mouse brains (Cheng et al., 2022).

### 2.4 X-linked intellectual disability

Intellectual disability (ID) is an early-onset neurodevelopmental condition characterized by deficits in intelligence (IQ < 70) and concomitant defects in adaptive behavior (Piton et al., 2013).

Intellectual disability is skewed toward males and has been linked to up to 100 ID-associated mutations on the X chromosome, characteristic of a subclass of ID called X-linked intellectual disability (XLID) (Wulff-Fuentes et al., 2021). A study revealed a missense mutation in the OGT catalytic domain in XLID patients. X-ray crystallography showed structural changes caused by this mutation, which reduced OGT activity. Mouse embryonic stem cells with the mutation had lower OGA and O-GlcNAc levels. This finding suggests a link between O-GlcNAcome alterations and intellectual disability in OGT mutation carriers (Pravata et al., 2020).

As mentioned above, another glycosylation mechanism, N-glycosylation, also plays a significant role in the pathogenesis of XLID through its specific enzymatic processes and associated genetic variations. N-linked glycosylation begins in the endoplasmic reticulum with the synthesis of a highly conserved dolichol-linked oligosaccharide precursor. The UDP-GlcNAc glycosyltransferase that catalyzes the second sugar addition of this precursor consists of at least two subunits, ALG14 and ALG13, in most eukaryotes (Averbeck et al., 2007). Pathogenic variants in ALG13 were first reported as X-linked causes of congenital disorders of glycosylation type 1 (ALG13-CDG) and as causes of XLID (Timal et al., 2012). A recent study provides evidence that ALG13 pathogenic variants can mildly alter N-linked protein glycosylation in both female and male individuals. Although the underlying mechanism remains unclear, these data enhance the understanding of the phenotypic heterogeneity caused by pathogenic variants in ALG13 (Alsharhan et al., 2021).

### 2.5 GM3 synthase deficiency

The ST3GAL5 gene encodes GM3 synthase, a sphingolipid-specific sialyltransferase (Schnaar, 2019). ST3GAL5 GM3 synthase deficiency (GM3SD) is caused by pathogenic mutations in this gene and manifests as neurodevelopmental disorders. Mutations in the ST3GAL5 gene cause a severe, autosomal recessive neurological disease that typically manifests in infancy and is characterized by progressive microcephaly, intellectual disability, dyskinetic movements, blindness, deafness, intractable seizures, and changes in pigmentation (Boccuto et al., 2014; Bowser et al., 2019; Fragaki et al., 2013; Gordon-Lipkin et al., 2018; Heide et al., 2022; Indellicato et al., 2019; Lee et al., 2016).

To investigate how the loss of GM3 affects neuro-specific glycolipidation, glycosylation, and cell signaling, Dookwah et al. (2023) analyzed the levels of O-GlcNAc in whole-cell lysates of induced pluripotent stem cells (iPSCs) and neural crest cells (NCCs) from patients with WT and GM3SD and assessed the fidelity of protein O-GlcNAcylation in the absence of complex gangliosides. A previous study has shown that in WT and GM3SD, the abundance of O-GlcNAc-modified proteins increases when iPSCs differentiate into NCCs. Compared with WT, the two variants of GM3SD, p.Glu355Lys and p.Arg288Ter, had comparable fold increases in O-GlcNAcylation in NCCs relative to iPSCs. When WT and GM3SD iPSCs differentiated into NCCs, the O-GlcNAcylation of the protein increased. These findings demonstrated that the O-GlcNAc of the protein was more widely distributed in GM3SD cells, as WT and GM3SD iPSCs differentiated into NCCs. These results may suggest a mechanism by which loss of GM3 affects neuro-specific glycolipid glycosylation (Dookwah et al., 2023).

Gangliosides are an important class of glycosylated glycolipids that are widely found in nerve cell membranes and play crucial roles in the normal function of nerve cells and the immune system (Cheon and Orsulic, 2011; Crocker et al., 2007; Eskandari-Sedighi et al., 2023; Smeekens and Kulis, 2021). Sialic acid in gangliosides plays an important role in neurological disorders by binding to the cell surface receptor Siglecs (sialic acid-binding immunoglobulin-like lectins). A study has systematically analyzed the binding properties of human (hSiglecs) and murine (mSiglecs) Siglecs to a variety of gangliosides via optimized liposome formulations. The results showed that while the binding properties of mSiglecs to most gangliosides were similar to those of hSiglecs, the binding properties to specific gangliosides (e.g., GM1a) were significantly different. This discrepancy suggests that the impact of species-specific differences on experimental results needs to be carefully considered when murine models are used for studies of human neurological disorders (Crocker et al., 2007; Schmidt et al., 2024).

## 3 Neurodegenerative disorders

**FIGURE 2:**
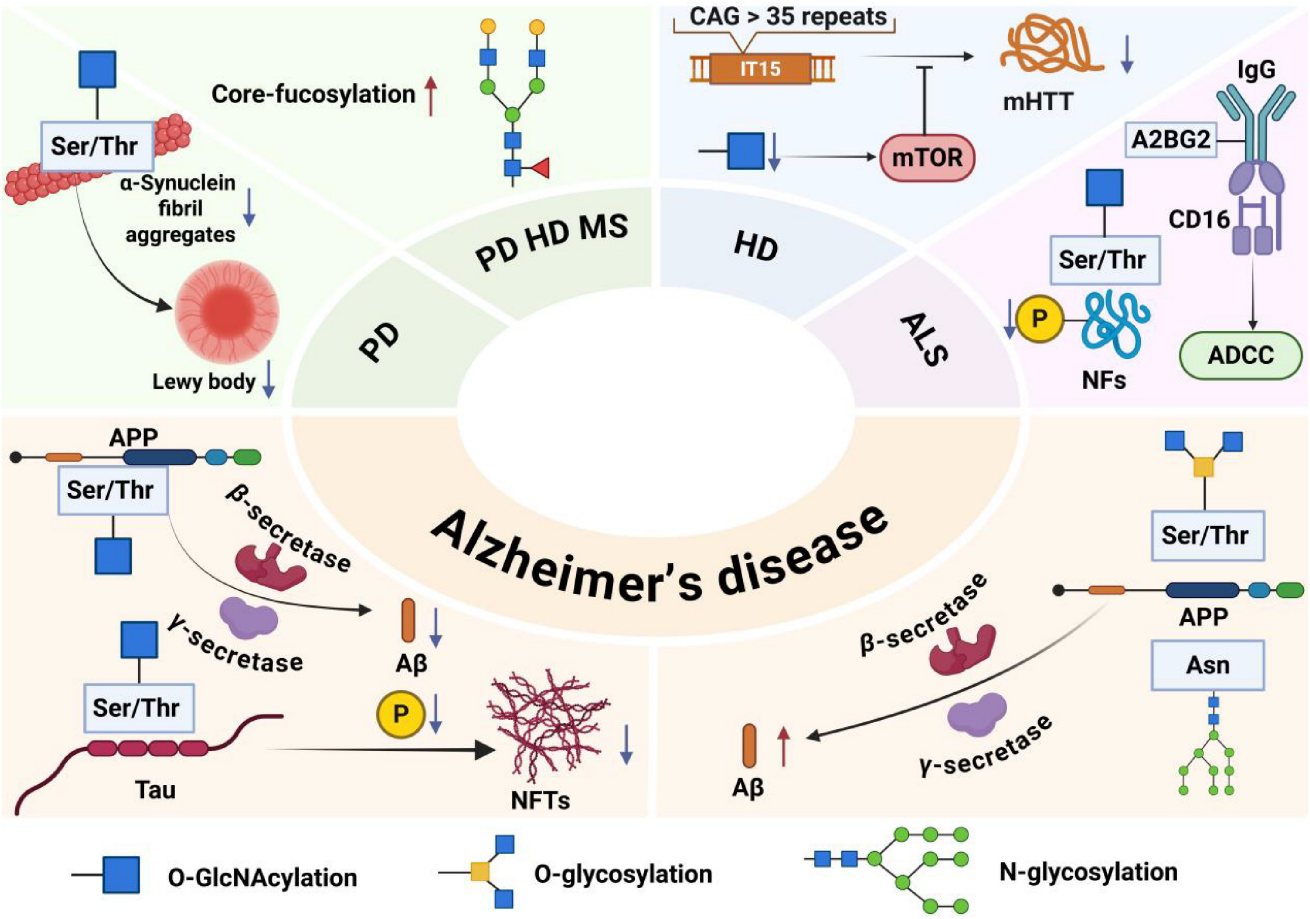
Pathogenic roles of major glycosylation types in neurodegenerative diseases. In Alzheimer’s disease, O-GlcNAcylation of amyloid precursor protein and Tau inhibits amyloid-β deposition and Tau hyperphosphorylation; N-glycosylation regulates amyloid precursor protein processing and neuroinflammation, while O-glycosylation promotes plaque formation. In Parkinson’s disease, O-GlcNAcylation prevents α-synuclein aggregation. In Huntington’s disease, it suppresses mutant huntingtin aggregation via mTOR. In ALS, O-GlcNAcylation modulates neurofilament phosphorylation, and specific N-glycans enhance antibody-dependent cytotoxicity. Elevated core-fucosylation disrupts protein function across PD, HD, and MS, contributing to pathogenesis and offering diagnostic value.

Neurodegenerative disorders are hereditary and sporadic conditions characterized by progressive dysfunction of the nervous system. These disorders are often associated with atrophy of the affected central or peripheral nervous system structures. Neurodegenerative diseases include Alzheimer’s disease, Parkinson’s disease, Huntington’s disease, multiple sclerosis, amyotrophic lateral sclerosis, etc. Neurodegeneration represents a core pathophysiological mechanism underlying several major brain diseases (Przedborski et al., 2003). It constitutes a major health problem characterized by synaptic and neural network dysfunction, often accompanied by the deposition of physiochemically altered protein variants in the brain (Kovacs, 2019; Lamptey et al., 2022). Age is the single greatest risk factor for all neurodegenerative diseases, but recent studies suggest that the combination of an individual’s genetic blueprint and environmental factors likewise increases the risk of neurodegenerative diseases. Furthermore, although the expression of specific genes (within individuals) has been linked to neurodegenerative diseases (Liu et al., 2022), the timing and extent of neurodegenerative diseases are largely dependent on their immediate environment (Jain and Chen-Plotkin, 2018; Jain et al., 2012). Notably, glycosylation in neurodegenerative diseases has gradually become a research hotspot (Figure 2).

### 3.1 Alzheimer’s disease

Alzheimer’s disease (AD) is one of the most common neurodegenerative diseases, with dementia as the main symptom, and tends to occur in middle-aged and elderly people. As of 2023, the latest statistics show that the incidence of Alzheimer’s disease has increased with the acceleration of global aging (Alzheimers and Dementia, 2023). It is characterized by amyloid plaques and neurofibrillary tangles (Rostagno, 2022).

The formation of amyloid plaque Aβ is closely related to the amyloid precursor protein APP, a widely present glycoprotein in the brain that plays an important role in nervous system development, helps regulate synaptic transmission, maintains calcium homeostasis, and plays a neuroprotective role (Murphy and LeVine, 2010). Aβ is formed from the amyloid precursor protein APP after cleavage by β-secretase and γ-secretase in sequence via the amyloidosis pathway. When the amyloidosis pathway of APP is upregulated, it eventually leads to increased Aβ production and promotes the onset of AD (Depp et al., 2023; Hefter et al., 2020).

Another pathological feature, neurofibrillary tangles, consists of paired helical filaments of hyperphosphorylated Tau protein. Tau, a microtubule-associated protein (MAP), is found in neurons of the central and peripheral nervous systems of vertebrates and is abundant in axons, helping to maintain the structure and function of microtubules (Scheltens et al., 2021). Tau, in its abnormally phosphorylated form, aggregates and accumulates in neurofibrillary tangles, leading to synaptic loss, neuroinflammation, and neurodegeneration, contributing to the onset of AD (Sun et al., 2021).

In addition, the onset of Alzheimer’s disease is also associated with the glycosylation of various proteins, including APP and Tau proteins (Zhao and Lang, 2023).

The pathogenesis of AD is closely related to O-GlcNAcylation, O-glycosylation, and N-glycosylation. O-GlcNAcylation is a protein glycosylation modification that adds O-GlcNAc to the serine or threonine residues of many proteins. This protein modification, which regulates protein transcription, translation, and protein deposition, occurs throughout the body and is especially common in the brain. The regulation of O-GlcNAc in the brain has been shown to alter synaptic and neuronal function and protect against a variety of neurodegenerative diseases (Du et al., 2024; Lee et al., 2021).

The regulation of O-GlcNAc can activate the non-amyloid pathway of APP, downregulate the amyloidosis pathway, reduce the production of Aβ, promote the cleavage of generated Aβ, prevent the subsequent nerve damage caused by the hyperphosphorylation of Tau induced by Aβ, reduce the number of tangles of Tau neurons modified by O-GlcNAc, and inhibit neurodegeneration (Akasaka-Manya and Manya, 2020).

O-linked glycosylation of APP induces a conformational change of APP, upregulates the amyloid pathway of APP, and eventually leads to an increase in amyloid plaques. Along with the gradual deposition of amyloid plaques in cerebrovascular and brain tissues, amyloid plaques damage cerebrovascular and brain tissues, resulting in functional impairment of the nervous system and ultimately leading to the occurrence of diseases (Singh et al., 2021).

Protein N-glycosylation is ubiquitous in the brain and is strongly associated with cognition and memory. Experiments have shown that protein N-glycosylation is involved in a variety of dysregulated processes and pathways in the AD brain, including extracellular matrix dysfunction, neuroinflammation, synaptic dysfunction, altered cell adhesion, lysosomal dysfunction, endocytic trafficking dysfunction, endoplasmic reticulum dysfunction, and cell signaling dysfunction (Zhang et al., 2020).

### 3.2 Parkinson’s disease

The essence of Parkinson’s disease (PD) is a neurodegenerative disease, and its pathogenesis is closely related to mitochondrial dysfunction, oxidative stress, α-synuclein aggregation, and abnormal glycosylation modification, pathological manifestations are characterized by early and significant death of dopaminergic neurons in the substantia nigra pars compacta (SNpc), resulting in classic Parkinson’s motor symptoms due to dopamine deficiency (Kalia and Lang, 2015).

O-linked N-acetylglucosaminylation protects neurons by inhibiting harmful aggregation and toxicity of alpha-synuclein. α-Synuclein is a small protein with 140 amino acids that is divided into three distinct regions: the positively charged N-terminal region, an amphiphilic helical structure that acts as a mitochondria-targeting sequence peptide and may be involved in mitochondrial dysfunction; a central hydrophobic region, with a high tendency to aggregate; and a highly acidic C-terminal domain, rich in acidic amino acid residues, which is key to the solubility and stability of alpha-SYN and can serve as a domain for interaction with other proteins. The N-terminal domain is the central hydrophobic region, which plays a key role in alpha-SYN fiber aggregation and the formation of Lewy bodies (Rocha et al., 2018).

Misfolding and abnormal aggregation of α-synuclein are important causes of degeneration of dopaminergic neurons, and in PD, the up-regulated O-GlcNAc level of α-synuclein can effectively inhibit its misfolding and aggregation, thereby preventing the harm caused by protein degeneration to neurons.

Through blood analysis, serum levels of N-glycan core fucosylation and sialylation increased in male patients with PD, which has important diagnostic significance for male patients with PD. The changes in sialylation and fucosylation levels were most significant in PD patients with triantennal and quadantennal glycans, which were derived mainly from male patients. These glycosylation changes are intricately linked to endoplasmic reticulum stress and the unfolded protein response, suggesting that N-glycan dysregulation may contribute to the protein aggregation and neuronal vulnerability central to PD pathogenesis (Rebelo et al., 2023). This alteration was also found to be critical in the control group and in the classification of men with Parkinson’s disease (Váradi et al., 2019).

### 3.3 Huntington’s disease

Huntington’s disease (HD) is an autosomal dominant inherited progressive neurodegenerative disorder with a distinct phenotype that includes chorea and dystonia, uncoordinated movements, cognitive decline, and behavioral difficulties. Normal Huntington protein (HTT) plays a variety of roles in cells, including vesicle transport, the transcription of neuronal genes, the production of brain-derived neurotrophic factor (BDNF), and the inhibition of apoptosis, protecting neurons from damage. The mutant Huntington’s protein in Huntington’s disease is derived from the repeated amplification of the CAG sequence, which produces a variable-length polyglutamine chain at the N-terminus (Walker, 2007), and the mutant Huntinting protein accumulates deposits in the striatum inside the brain, damaging neurons in the striatum and leading to neuronal apoptosis and striatum atrophy.

An important pathogenic mechanism of Huntington’s disease is the abnormal aggregation of Huntington’s protein. A previous study revealed that, in neuroblastoma cells and fruit fly models of Huntington’s disease, O-GlcNAc modification levels are reduced, whereas autophagosome and lysosome functions are enhanced, enabling increased basal autophagy to clear accumulated toxic Huntington’s proteins. In a neuroblastoma cell model, lowering O-GlcNAc modification activates the mTOR signaling pathway, which promotes autophagy and clears the Huntington protein. In summary, modulating the level of O-GlcNAc modification may be a potential treatment for Huntington’s disease (Ryan et al., 2019).

In addition, MS analysis of total glycans in the brain tissue and serum of transgenic mouse models of HD showed N-sugar chains associated with core fucosylation and double-branched mannosidic acid types were expressed at increased levels in the brains of HD transgenic mice. The N-sugar chain structure of core fucosylation and double-branched mannosidic acid is prevalent in nerve cells, but their increase may indicate dysfunction or abnormality in nerve cells while reflecting abnormalities in the protein glycosylation pathway in Huntington’s disease, which may lead to impaired nerve cell function. These changes in N-glycosylation modifications may be potential biomarkers for the diagnosis and treatment of HD (Gizaw et al., 2015).

### 3.4 Multiple sclerosis

Multiple sclerosis (MS) is a chronic autoimmune disease of the CNS. The early pathological features are inflammatory lesions around small veins, which gradually lead to oligodendrocyte injury, demyelination, and eventually irreversible axon injury. The specific pathogenesis is still not fully understood (Dobson and Giovannoni, 2019; Oh et al., 2018).

Multiple sclerosis is a neurodegenerative disease of the CNS, and immune cells are involved in the disease progression of MS. For example, CD4+ T cells secrete a variety of inflammatory cytokines, leading to severe inflammation and damage to myelin and neurons, causing multiple sclerosis (Chien et al., 2018). Core fucosylation is a modification of fucosylation in the form of an α 1,6 bond to N-acetyl-glucosamine (GlcNAc), the innermost part of N-glycan. It involves structure-specific N-glycosylation. Dysregulation of total N-glycan expression and branching induces changes characteristic of inflammatory damage, which destroys the insulation around nerve fibers through abnormal functioning of the interface between immune cells, CNS neurons, and oligodendrocytes (Cvetko et al., 2020).

### 3.5 Amyotrophic lateral sclerosis

Amyotrophic lateral sclerosis (ALS) is a rapidly progressive motor neuron neurodegenerative disease that clinically manifests as localized attacks of muscle weakness and progresses rapidly. The specific etiology of ALS remains an active area of investigation, with current research indicating that both genetic and environmental factors significantly contribute to its development. Notable risk factors include genetic mutations, exposure to heavy metals, smoking, and a sedentary lifestyle. Prolonged exposure to these risk factors disrupts protein homeostasis in neuronal cells, leading to mitochondrial dysfunction and cellular inflammatory responses, ultimately resulting in neuronal apoptosis (Feldman et al., 2022).

In the neurodegenerative disease ALS, many proteins are found to be hyperphosphorylated, including neurofilament proteins (NFs).

Neurofilament proteins are composed of different types of monomers, including a serine- and threonine-rich head (N-terminal), a region body composed of a highly conserved amino acid sequence formed by an alpha helix, and a highly variable tail (C-terminal). It plays an important role in the development of the nervous system. It can participate in the formation of the cytoskeleton, maintain good elasticity of nerve fibers, maintain radial growth of axons and nerve conduction velocity, regulate the fixation and function of organelles, and regulate the function of postsynaptic membrane receptors (Yuan et al., 2017). In the ALS mouse model, O-GlcNAc levels are significantly reduced, and the ability to inhibit neurofilament protein phosphorylation is weakened, ultimately leading to neurodegeneration (Shan et al., 2012).

The pathogenic mechanism of ALS is also closely related to serum glycoprotein IgG, whose formation is influenced mainly by sialylation or core fucosylation, which is a special N-glycan. Upon analysis of the Fc N297-glycans of IgG in the serum of ALS patients, a distinct glycan, A2BG2, was identified. This glycan increases the binding affinity of IgG for CD16 on effector cells, thereby enhancing antibody-dependent cellular cytotoxicity (ADCC) (Borrok et al., 2012). It has been demonstrated that intact ALS-IgG enhances effector cell activation and ADCC-responsive neuronal damage, leading to neurodegenerative disease (Costa et al., 2019).

## 4 Neuropsychiatric disorders

Neuropsychiatric disorders cover a wide range of complex conditions characterized by deficits in cognitive functioning, psychological disturbances, and somatic symptoms. These disorders include but are not limited to schizophrenia, depression, posttraumatic stress disorder (PTSD), and major depressive disorder (MDD), each with its own set of challenges (Cheng et al., 2021).

**FIGURE 3:**
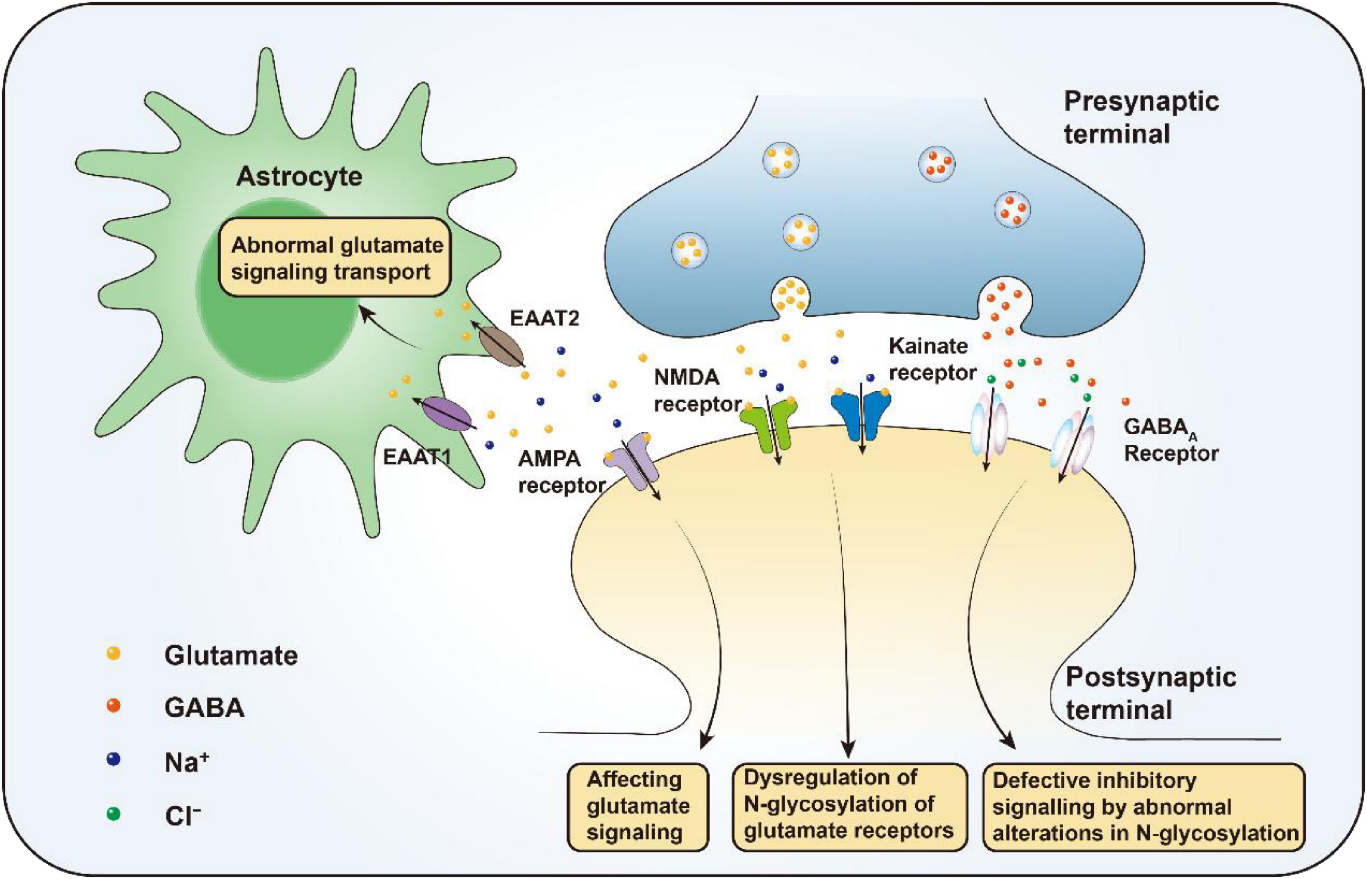
Glycosylation-mediated mechanisms in neuropsychiatric disorders. In schizophrenia, aberrant N-glycosylation of glutamate transporters (EAAT1/2) and receptors (AMPA, NMDA, kainate) disrupts glutamate signaling, while altered GABAA receptor glycosylation impairs inhibitory transmission. Epigenetic regulation of FUT8 influences N-glycan levels linked to PTSD. Altered N-glycan profiles promote inflammation in major depressive disorder (MDD), and disrupted O-GlcNAcylation impairs astrocyte metabolism, affecting energy supply and fear memory, thereby increasing MDD susceptibility.

The cause of mental illness is largely attributable to genetic factors (Uher and Zwicker, 2017) with thousands of genetic variants involved in the development of risk for most mental disorders (Dudbridge, 2013; Wray et al., 2014). Approximately two-thirds of the genetic associations are associated with schizophrenia, bipolar disorder, and major depressive disorder. Moreover, the most common and rarest genetic variants are not associated with a range of psychiatric disorders (Cross-Disorder Group of the Psychiatric Genomics Consortium et al., 2013; Cross-Disorder Group of the Psychiatric Genomics Consortium, 2013). In addition, a variety of environmental factors that influence the development of neuropsychiatric disorders, including pregnancy risk factors (e.g., infections, malnutrition, and heavy metals) and perinatal risk factors (e.g., prematurity, the season of birth, and delivery complications), the childhood environment, and adolescent drug use (Arseneault, 2017; Davis et al., 2016; Rai et al., 2012; Vassos et al., 2016). Dysregulation of copper homeostasis may also contribute to severe mental disorders (Manchia et al., 2024). The increasing focus on glycosylation as a research topic has implications for the pathogenesis of neuropsychiatric disorders (Figure 3).

### 4.1 Schizophrenia

Schizophrenia is a heterogeneous psychiatric disorder strongly associated with glutamate. Many studies exploring the etiology of schizophrenia have shown that the occurrence of schizophrenia is associated with abnormal glycosylation and that some receptors involved in glycosylation, such as AMPA and kainate receptor subunits, the glutamate transporters EAAT1 and EAAT2, and the Gamma-aminobutyric acid type A (GABAA) receptor, play important roles in this process.

Numerous studies have demonstrated brain region- and subunit-specific abnormalities in the expression of subunits of the AMPA subtype of glutamate receptors in schizophrenia. There is evidence suggesting that abnormalities in the expression of proteins are related to AMPA receptors. AMPA receptors are responsible for primary depolarization during glutamate-mediated neurotransmission. Disturbances of glutamate neurotransmission, particularly the underactivity of glutamate receptors, have been hypothesized to contribute to the pathophysiology of schizophrenia. In addition, glutamate receptor subunits in schizophrenia exhibit bidirectional changes in glycosylation, with GluK 2 having more high-mannose N-glycans, whereas GluA 2 has fewer. GluA2 subunits isolated from individuals with schizophrenia exhibit decreased binding to ConA, a lectin that binds high levels of mannose to complex with N-glycans (Maupin et al., 2012), which impairs the protein transport of the subunits and disrupts the formation of AMPA receptor complexes, thereby affecting global glutamate signaling (Tucholski et al., 2013a).

The NMDA and kainate receptors are multimeric complexes comprised of multiple subunits. NMDA receptors are formed from a combination of GluN1, GluN2A-D, and GluN3A-B subunits, while kainate receptors are assembled from GluK1-GluK5 subunits (Paoletti et al., 2013). The majority of these subunits are subject to extensive N-glycosylation, a critical post-translational modification that regulates their trafficking, stability, and function (Kaniakova et al., 2016; Vernon et al., 2017). Endoglycosidase-H (EndoH) is a recombinant glycosidase capable of cleavage of the high mannose in the N-glycoprotein and the chitobiose core structure of some heterozygous oligosaccharides, removing the N-linked high mannose from the glycoprotein. GluN1, GluN2A, GluN2B, GluK2 and GluK5 are all sensitive to EndoH (Tucholski et al., 2013b). The GluK2 kainate receptor subunit in schizophrenia showed higher sensitivity to EndoH, indicating the presence of high-mannose/hybrid-type N-glycans in the disease state. Global dysregulation of the N-glycosylation of glutamate receptors is an important cause of schizophrenia (Williams et al., 2020). GABAA receptors cause schizophrenia through a similar mechanism. GABAA receptors are isolated from the superior temporal gyrus in schizophrenia. The enzymatic deglycosylation and lectin affinity analysis revealed the presence of N-glycans on the α1, α4, β1, β2, and β3 subunits (Mueller et al., 2014). Each subunit displays different changes in glycosylation, with the α1 subunit showing decreased high-mannose N-glycans, the β1 subunit showing increased high-mannose N-glycans, and the β2 subunit showing increased total N-glycosylation. This abnormality of altered glycosylation may contribute to the inhibitory signaling defects observed in schizophrenia (Mueller et al., 2015).

EAAT 1 and EAAT 2 are excitatory amino acid transporters that do not differ in high-mannose N-glycans, but their complex N-glycans in schizophrenia are distinct from those in the normal human brain. There are fewer complex N-glycans in EAAT1 and EAAT2 in patients with schizophrenia than in healthy individuals (Bauer et al., 2010). Conduction of glutamate signaling is affected. These findings suggest that abnormal N-glycosylation of EAAT 1 and EAAT 2 in schizophrenia leads to the abnormal glutamate signaling transport, which contributes to the development of schizophrenia.

### 4.2 Posttraumatic stress disorder

Posttraumatic stress disorder refers to a mental disorder that is delayed or persistent after severe and intense mental attack, such as brutal war, strong earthquakes, terrorist scenes, murder scenes, vicious traffic accidents, or violent violence. Many factors cause PTSD, including psychological, genetic, and environmental factors, and recent studies have shown that changes in glycosylation can also have an impact on PSTD. Fucosyltransferase 8 (FUT8) is the enzyme that catalyzes the transfer of fucose to the innermost GlcNAc of N-glycans, a process known as core fucosylation. Mutations in the FUT8 gene are associated with defective glycosylation and various pathological abnormalities (Tudor et al., 2023). By analyzing the molecular links between plasma N-glycan levels, different genetic polymorphisms located in the FUT8 linkage region, and PTSD, studies have shown that plasma N-glycan levels are significantly associated with PTSD and with the rs6573604, rs11621121, rs10483776 and rs4073416 polymorphisms. Among them, the rs6573604 T allele had the greatest effect on N-glycosylation levels (Tudor et al., 2023).

The T allele of the rs6573604 polymorphism is closely related to a higher risk of PTSD, as well as lower levels of tetra-antenna, tetragalactosylated and tetrasialylated (A4G4S4) N-glycans in plasma. Elevated levels of highly sialylated tri- and tetra-antennary N-glycans often reflect persistent inflammation (Liu et al., 2007; Higai et al., 2005). Because the rs6573604 polymorphism is located within the microRNA 4708 gene (MIR4708), which is near the 50th end of the FUT 8 gene, it may affect the plasma levels of these N-glycans through molecular epigenetic mechanisms (Tudor et al., 2023).

### 4.3 Major depressive disorder

Major depression is a common mental disorder that is characterized by mood changes and a high suicide rate. At present, the incidence of major depression is increasing worldwide, which has a certain impact on the development of society. There have been studies on the pathogenesis of MDD. It was recently found to be closely associated with abnormalities in N-glycosylation and O-GlcNAcylation.

The experience of chronic and traumatic stressors has long been recognized as a major risk factor for the development of depression. However, it is currently believed that inflammation plays a role in depression, and the occurrence of inflammation is associated with altered N-glycosylation levels. There are two high levels of inflammatory substances in depression: IL-6 and C-reactive protein (CRP). IL-6 and CRP levels are associated with specific N-glycan profile alterations (Boeck et al., 2018). Alterations in the N-glycan profile lead to the occurrence of inflammation, which is currently an important direction for studying the etiology of depression.

Astrocyte dysregulation in the medial prefrontal cortex (mPFC) has been implicated in various neuropsychiatric disorders, such as MDD (Ongür et al., 1998). Astrocytic O-GlcNAc transferase enzyme (OGT) can influence the expression of proteins in the mPFC, thus affecting metabolic and biosynthetic processes. It has been proven that astrocytes produce lactate and ATP to modulate mood disorders and fear memory (Cao et al., 2013; Kambe et al., 2021; Yin et al., 2021). Glucose is the major energy source for astrocytes. In glucose metabolism, in addition to generating ATP and lactate, some glucose is used for the biosynthesis of UDP-N-acetylglucosamine (UDP-GlcNAc), which serves as a donor molecule for O-GlcNAcylation. This metabolic pathway is modulated by both O-GlcNAc transferase enzyme (OGT) and O-GlcNAcase (OGA) (Fan et al., 2021). When O-GlcNAcylation is dysregulated by OGT and OGA, the metabolic processes in astrocytes become disturbed, which affects the metabolism of ATP and lactate (Yang and Qian, 2017). As a result, mood disorders and fear memory are not regulated, and the probability of MDD increases.

Major depressive disorder and PTSD exhibit high comorbidity rates, a phenomenon increasingly attributed to shared biological pathways–including dysregulated neuroimmune responses and synaptic dysfunction (Daskalakis et al., 2024). Mendelian randomization analyses indicate that genetically determined depressive phenotypes exert a causal influence on PTSD, while shared genetic evidence suggests that PTSD may represent a subtype of depression (Zhang F. et al., 2022). Notably, glycosylation, as a critical post-translational modification, plays a key regulatory role in both of these pathogenic mechanisms. Genome-wide association studies have revealed a causal relationship between IgG N-glycan profiles and psychiatric disorders, in which IGP7 demonstrates a protective effect against major depressive disorder, while elevated levels of IGP22 are associated with an increased risk of PTSD (Lv et al., 2024). Given that glycosylation serves as a fundamental mechanism for modulating protein function, it represents a plausible, yet not fully explored, molecular link for future research into MDD-PTSD comorbidity.

## 5 Epilepsy

Epilepsy is a brain disorder characterized by recurrent seizures due to brief abnormalities in neuronal activity. Comorbidities such as ID, ASD, and other psychiatric symptoms may develop before or after epilepsy onset (Caraballo et al., 2014; Ragona et al., 2011).

Genetic factors contribute to approximately 50% of cases, with core pathogenic variants concentrated in genes encoding ion channels (SCN1A, KCNQ2, KCNT1) and neurotransmitter receptors (GABRA1, GRIN2A) that regulate neuronal excitability and synaptic transmission (Dhiman, 2017; Hansen et al., 2021; Shah and Aizenman, 2014). These variants are classified as gain-of-function (GoF) or loss-of-function (LoF), which disrupt neuronal excitability. For example, sodium or potassium channel GoF enhances hyperexcitability, and GABA_*a*_ receptor LoF weakens inhibitory. The core of the pathogenesis is an imbalance of excitability-inhibition in the brain, where pathogenic mutations disrupt the normal function of ion channels or neurotransmitter receptors, leading to abnormal, excessive, or synchronized electrical activity in neurons, ultimately leading to seizures (Allen et al., 2020).

In addition, SLC35A2, which encodes the UDP-galactose transporter involved in glycosylation, also plays a role in the pathogenesis of epilepsy (Elziny et al., 2023). In patients with MOGHE (mild cortical dysplasia with oligodendrogliosis and epilepsy), somatic mutations in the SLC35A2 gene result in loss of UDP-galactose transporter function (Kodera et al., 2013). This deficiency directly impairs N-glycan synthesis, which causes glycan truncation and increased levels of agalactosylated glycoforms. As a result, glycoproteins lacking galactosylation are significantly enriched in pathways related to cell adhesion and axon guidance. Disruption of these pathways, which are critical for normal neuronal migration, positioning, and synaptic connections, leads to cortical development abnormalities. Furthermore, glycosylation abnormalities may alter the function and localization of neuronal surface receptors and ion channels, which affects the electrophysiological properties of neurons, leading to abnormal discharges and serving as the basis for epileptic seizures (Liu et al., 2025).

The polypeptide N-acetyl-D-galactosamine-transferase 2 isoenzyme encoded by GALNT2 is a key initiator of this type of O-glycosylation (Schjoldager et al., 2015). Its mutations directly block the assembly of O-glycans on target proteins, thereby impairing the function of glycoproteins involved in cell adhesion and axon guidance (e.g., phospholipid transfer protein, PLTP). This impairment leads to abnormalities in neuronal migration, localization, and synaptic connectivity, and also alters the localization and function of neuronal surface receptors (e.g., GABAA receptors) and ion channels–ultimately disrupting the excitatory-inhibitory balance in the brain and triggering seizures (Zilmer et al., 2020).

Furthermore, in temporal lobe epilepsy, the expression of OGT is reduced in the hippocampus, leading to a significant decrease in global O-GlcNAcylation levels. This leads dysregulation of synaptic transmitter transport and signaling mediated by synapse-associated proteins (e.g., Sortilin-Related Receptor, SORL1), impairing synaptic inhibition and indirectly enhancing neuronal excitability. The Tmod2 protein, which regulates the neuronal cytoskeleton, exhibits reduced O-GlcNAcylation and fails to maintain normal cytoskeletal architecture, thereby exacerbating the propensity for abnormal neuronal firing. O-GlcNAcylation dynamically modulates the localization and activity of neuronal surface ion channels and neurotransmitter receptors; its loss leads to abnormal ion channel excitability, ultimately disrupting the excitation-inhibition balance in the brain (Sánchez et al., 2019).

As discussed above, among the pathological features of epilepsy, O-glycosylation deficiency, N-glycan truncation, and altered O-GlcNAc levels can serve as distinct potential diagnostic biomarkers to aid in the diagnosis of epilepsy, while targeting glycosylation-related enzymes may represent a novel direction for epilepsy treatment.

## 6 Conclusion and perspectives

Glycosylation, a fundamental post-translational modification, critically regulates protein structure, function, and stability within the nervous system, exerting essential influence over neurodevelopmental processes, synaptic function, and neurotransmitter dynamics. The three principal types–N-glycosylation, O-glycosylation, and O-GlcNAcylation–demonstrate significant involvement in the pathogenesis of diverse neurological disorders. Evidence links disruptions in N-glycosylation and O-GlcNAcylation to the onset and progression of neurodevelopmental conditions such as ASD, ADHD, XLID, RTT, and GM3SD. In neurodegenerative diseases including AD, PD, HD, MS, and ALS, dysregulated glycosylation modulates key pathological features like amyloid-β deposition, Tau hyperphosphorylation, and α-synuclein aggregation, thereby directly influencing disease mechanisms. Furthermore, aberrant glycosylation patterns contribute to neuropsychiatric disorders (e.g., schizophrenia, MMD, PTSD) by impacting critical pathways including glutamatergic signaling, neuroinflammation, and brain energy metabolism involving ATP and lactate.

The correlation between particular glycosylation pathways and the etiology of neurological diseases underscores the promise for therapeutic innovation in this domain. A comprehensive exploration is imperative to elucidate the precise molecules of diverse glycosylation modifications that underpin specific disease processes within various neurological contexts. Recent advancements in targeted glycosylation therapeutics have demonstrated notable progress, particularly within the field of tumor immunotherapy. A groundbreaking innovation in this area is the deglycosylation-targeting chimera (DGlyTAC) platform, which enables precise removal of N-glycans from specific membrane proteins–such as the immune checkpoint molecules PD-L1 and CD47–via fusion of peptide N-glycosidase F (PNGase F) with targeted nanobodies or affinity bodies, thereby disrupting their functional interactions. Accumulating evidence indicates that DGlyTAC effectively impedes PD-L1/PD-1 binding and exhibits superior antitumor efficacy coupled with reduced toxicity compared to conventional antibody therapies in murine tumor models (Li et al., 2025). In parallel, glycosylation-targeted strategies are emerging as promising therapeutic avenues for neurological disorders. For instance, oral administration of N-acetylglucosamine has been shown to modulate N-glycan branching on immune cells, attenuate pro-inflammatory cytokine release, and reduce serum levels of neurofilament light chain (sNfL), a biomarker of neuroaxonal injury. Clinical observations further suggest potential improvement in neurological disability among multiple sclerosis patients following N-acetylglucosamine supplementation (Sy et al., 2023). Despite these promising developments, the therapeutic targeting of glycosylation in neurological diseases remains largely unexplored. There is a pressing need to enhance the sensitivity and resolution of glycomic detection tools to elucidate glycosylation patterns within the central nervous system under both physiological and pathological conditions. Addressing these fundamental issues is imperative for translating glycobiological findings into effective diagnostic and therapeutic approaches for neurological disorders.
